# MicroRNAs: From Mechanism to Organism

**DOI:** 10.3389/fcell.2020.00409

**Published:** 2020-06-03

**Authors:** Philipp J. Dexheimer, Luisa Cochella

**Affiliations:** Research Institute of Molecular Pathology (IMP), Vienna BioCenter (VBC), Vienna, Austria

**Keywords:** miRNA, evolution, development, Argonaute, Drosha, Dicer, biogenesis, silencing

## Abstract

MicroRNAs (miRNAs) are short, regulatory RNAs that act as post-transcriptional repressors of gene expression in diverse biological contexts. The emergence of small RNA-mediated gene silencing preceded the onset of multicellularity and was followed by a drastic expansion of the miRNA repertoire in conjunction with the evolution of complexity in the plant and animal kingdoms. Along this process, miRNAs became an essential feature of animal development, as no higher metazoan lineage tolerated loss of miRNAs or their associated protein machinery. In fact, ablation of the miRNA biogenesis machinery or the effector silencing factors results in severe embryogenesis defects in every animal studied. In this review, we summarize recent mechanistic insight into miRNA biogenesis and function, while emphasizing features that have enabled multicellular organisms to harness the potential of this broad class of repressors. We first discuss how different mechanisms of regulation of miRNA biogenesis are used, not only to generate spatio-temporal specificity of miRNA production within an animal, but also to achieve the necessary levels and dynamics of expression. We then explore how evolution of the mechanism for small RNA-mediated repression resulted in a diversity of silencing complexes that cause different molecular effects on their targets. Multicellular organisms have taken advantage of this variability in the outcome of miRNA-mediated repression, with differential use in particular cell types or even distinct subcellular compartments. Finally, we present an overview of how the animal miRNA repertoire has evolved and diversified, emphasizing the emergence of miRNA families and the biological implications of miRNA sequence diversification. Overall, focusing on selected animal models and through the lens of evolution, we highlight canonical mechanisms in miRNA biology and their variations, providing updated insight that will ultimately help us understand the contribution of miRNAs to the development and physiology of multicellular organisms.

## Introduction

### The Emergence of Small RNA-Guided Effector Systems

Regulation of gene expression by small RNAs emerged as an ancient feature of cellular biology and is found in all three domains of life (bacteria, archaea and eukarya). Having evolved primarily as a means of defense against foreign nucleic acids (Shabalina and Koonin, 2008; Obbard et al., 2009) the principle of small RNAs specifically guiding effector proteins to selected nucleic acids via antisense-complementarity is a recurrent theme in biology. At their core, these RNA-based interference (RNAi) systems consist of two components, a nucleic acid allowing for sequence-specific target recognition, and an effector protein that mediates downstream effects with varying outcomes.

In eukaryotes, the effector proteins that mediate the silencing of target nucleic acids are part of the Argonaute protein family. After their origin in prokaryotes (Makarova et al., 2009; Olovnikov et al., 2013; Swarts et al., 2014a, b; Willkomm et al., 2015), Argonautes diversified into a versatile class of effector proteins, forming the core of various multiprotein regulatory systems or RNA-Induced Silencing Complexes (RISC). They all share common structural elements and the ability to bind short, single stranded RNAs in a conformation that enables base pairing with target RNAs (Steiner et al., 2007; Farazi et al., 2008; Takeda et al., 2008; Czech and Hannon, 2010). The other critical protein component of eukaryotic RNA-induced silencing pathways are nucleases that process precursor RNAs into small RNAs that can be loaded onto Argonaut proteins. A major player in multiple RNAi pathways is Dicer, an RNAse III type endonuclease that cleaves double-stranded RNA molecules to generate targeting-competent small RNAs that guide the effector machinery. Although no prokaryotic homolog of Dicer has been found to date, the origins of individual domains can be traced back to prokaryotes (Shabalina and Koonin, 2008). Together, RNase III type endonucleases and Argonaute proteins lie at the heart of diverse small RNA based pathways involved in multifaceted aspects of molecular biology.

**FIGURE 1:**
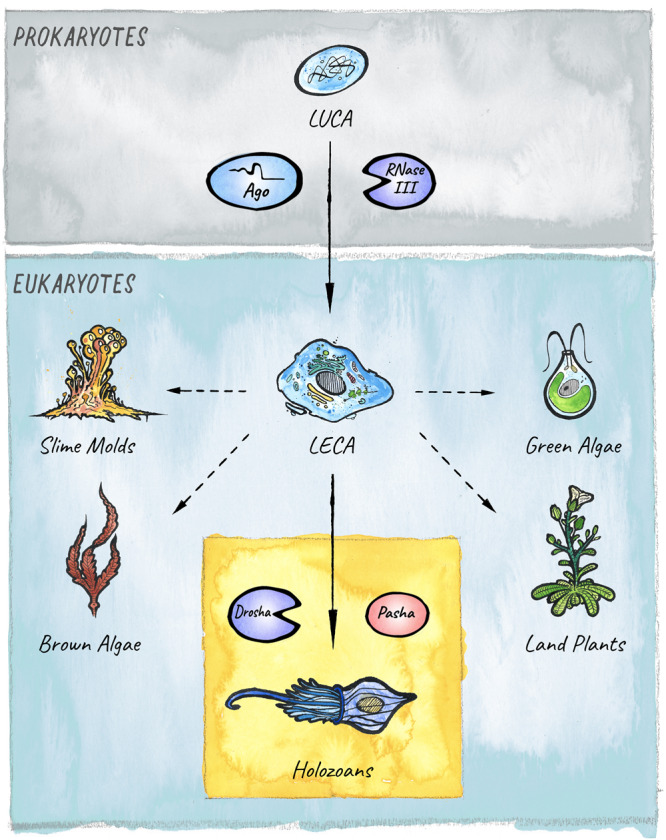
The evolutionary origins of miRNAs in eukaryotes. Two key players in small RNA-mediated silencing, Argonaute proteins and RNase III like enzymes, originated in prokaryotes. The miRNA pathway in animals emerged with the birth of the Microprocessor, composed of Drosha and Pasha, in unicellular holozoans (Bråte et al., 2018). Diverse lineages that branched from the last eukaryotic common ancestor (LECA) also evolved miRNA-like pathways. However, it is still under debate whether these evolved independently in four additional clades: Slime molds, Green algae, Brown Algae, and Land plants (Kruse et al., 2016; Valli et al., 2016; Cock et al., 2017; Bråte et al., 2018); or if the pathway was already present in the last common ancestor (Moran et al., 2017).

The different modules that constitute the eukaryotic RNAi systems likely originated in archaea, bacteria, and bacteriophages (Koonin, 2017). This core structure has diversified, specialized, and acquired new functions in the course of eukaryotic evolution. A key innovation in diverse lineages was the ability to not only load and target foreign or parasitic RNAs, but also to feed endogenously produced RNAs into the existing RNAi pathways to achieve gene regulation. This formed the basis for the emergence and expansion of the endogenous class of small RNAs called microRNAs (miRNAs), whose regulatory potential has been harnessed in animals, plants, and other eukaryotic lineages for the establishment of elaborate gene regulatory networks that control development and physiology (Figure 1).

### What Is the Purpose of This Review?

The complexity of multicellular organisms relies on the segregation of functions across many distinct cell types, which requires intricate gene regulatory mechanisms. As versatile repressors of gene expression, miRNAs are thought to facilitate the generation of different cell types with highly specialized physiology (Alberti and Cochella, 2017). It comes as no surprise that the evolution of multicellular organisms has been accompanied by an increase in complexity of the miRNA pathway and the miRNA repertoires (Hertel et al., 2006; Heimberg et al., 2008; Wheeler et al., 2009; Berezikov, 2011; Guerra-Assunção and Enright, 2012; Hertel and Stadler, 2015). The use of this increased diversity of repressors has been exploited by the addition of multiple mechanisms that control which miRNAs are produced at specific times and in specific cell types; at what levels these accumulate to act effectively; and what the molecular consequences of their repression are.

A comprehensive understanding of the contributions of miRNAs thus requires bridging biochemical and mechanistic insights with the organismal level. When and where does miRNA-mediated regulation take place? How is the production of a specific miRNA controlled in space and time to achieve that? And how do miRNAs integrate into cellular gene regulatory networks that control development and physiology? Recent technological advances have enabled more quantitative assessment of the kinetics of miRNA production and turnover, and context-dependent differences in composition of miRISC during distinct developmental stages. This allows a more nuanced view of when, where and how miRNAs are utilized within animal development and post-developmental processes. Moreover, the sequencing of an increasing number of genomes has upgraded our ability to place miRNAs and the associated protein machinery into an evolutionary context. These layers of understanding will lead us to broader concepts of what miRNAs contribute to the phenotypic complexity observed in present-day organisms.

We do not attempt to provide a comprehensive survey of all the miRNAs that have been studied and their functions in different animals, for which we suggest the following starting points (Chen et al., 2014; Alberti and Cochella, 2017; Bartel, 2018). Instead, we emphasize general concepts from diverse mechanistic studies to understand how multicellular organisms have exploited the potential of this broad class of repressors. We first focus on how miRNA biogenesis is controlled and discuss how the different modes of regulation can affect miRNA function within an animal. Next, we discuss how different composition of the RISC complex and different cellular contexts can result in distinct outcomes of miRNA-mediated repression. Finally, we present an updated overview of how the miRNA repertoire has evolved and what this teaches us so far about how miRNAs have acquired functionality during evolution.

### Evolutionary Origins of the miRNA Pathway

In 1993, the product of the *Caenorhabditis elegans* gene *lin-4* was surprisingly found to give rise to a small non-coding RNA, processed from a longer hairpin precursor, which functions by repressing protein production from the *lin-14* mRNA via an antisense RNA–RNA interaction (Lee et al., 1993; Wightman et al., 1993). First thought of as a nematode peculiarity, it became clear in the early 2000s that such endogenous small RNAs like *lin-4*, in particular *let-7*, play a fundamental role across eukaryotic lineages (Pasquinelli et al., 2000; Reinhart et al., 2000). The discovery of *lin-4* and *let-7*, followed by the identification of hundreds of endogenous small RNAs derived from hairpin precursors in animal genomes, laid foundation to the miRNA field (Lee and Ambros, 2001; Lau et al., 2001; Lagos-Quintana et al., 2003; Aravin et al., 2003; Lim et al., 2003). Not only is the protein machinery associated with miRNAs highly conserved, but so are many miRNAs – with >30 miRNAs shared across all bilaterian animals, and hundreds of others conserved within specific clades. Moreover, at least 37% of *Drosophila* and 60% of human protein-coding transcripts are subjected to selective pressure to retain miRNA binding sites (Friedman et al., 2008; Agarwal et al., 2018), underscoring the biological significance of miRNA-mediated gene silencing.

**FIGURE 2:**
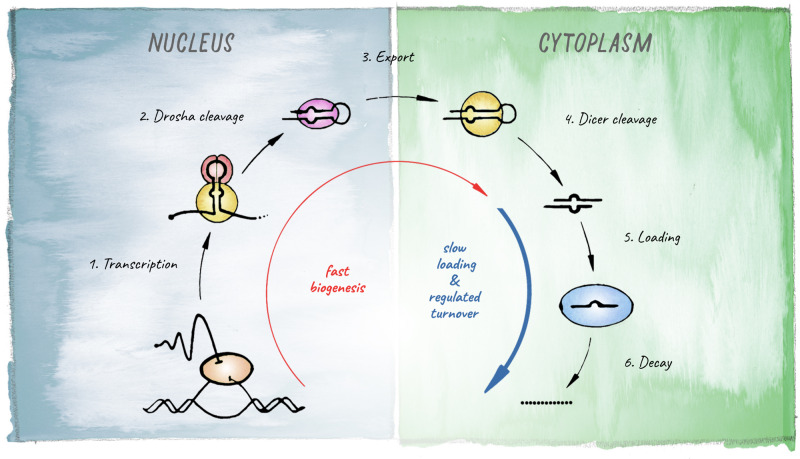
Schematic of the miRNA biogenesis pathway. The different steps leading to production of a mature miRNA are shown. Kinetic studies in *Drosophila* revealed that biogenesis is fast for most miRNAs but loading into Argonaute represents the rate-limiting step (based on Reichholf et al., 2019).

miRNAs are 21–23 nt small RNAs that elicit post-transcriptional downregulation of protein output from their target mRNAs. This is typically achieved through a combination of translational inhibition and promotion of mRNA decay (Jonas and Izaurralde, 2015; Bartel, 2018). The origin of miRNA-biology lies in the evolution of a mechanism that allowed for processing of long endogenous transcripts into short RNA duplexes that are further recognized and cleaved by Dicer. In animals this is achieved by the Microprocessor complex, composed of the RNase III endonuclease Drosha and its co-factor Pasha (Partner of Drosha, or DGCR8 in vertebrates). Cleavage of a primary miRNA transcript by the Microprocessor in the nucleus releases a hairpin precursor, that upon export to the cytoplasm is further cleaved by Dicer to give rise to a short RNA duplex with characteristic 2-nt 3′ overhangs. From this duplex, one strand is preferentially loaded into an Argonaute protein to generate a functional miRNA-induced silencing complex (miRISC) (Figure 2).

Drosha and Pasha/DGCR8 originated in unicellular holozoans after fungi branched from the pre-metazoan lineage (Bråte et al., 2018), placing the appearance of miRNAs before the onset of multicellularity. However, plants do not encode a homolog of Drosha or Pasha but process primary miRNA transcripts via the Dicer-homolog DCL1 (Dicer like 1), which generates mature miRNAs according to a similar principle involving two sequential endonucleolytic cleavage events (Voinnet, 2009). This led to the initial hypothesis that miRNAs evolved independently in animals and land plants. However, the possibility that the last common ancestor of these lineages might have already employed a common miRNA-like pathway for post-transcriptional regulation is not to be dismissed (Moran et al., 2017). Nevertheless, after their introduction, the number of miRNAs continuously expanded during the course of evolution, providing multicellular organisms with a diversified toolset for regulation of gene expression.

### miRNAs Are Essential for Development

Regulation by miRNAs evolved into a key feature of multicellular organisms. As general repressors of gene expression, miRNAs have been incorporated into gene regulatory networks that control diverse developmental and post-developmental processes. No higher metazoan lineage has tolerated the loss of essential miRNA pathway components. In fact, defects in the miRNA biogenesis machinery or the effector silencing factors results in severe embryogenesis defects in every animal studied.

In *C. elegans*, removal of the miRNA-specific Argonautes *alg-1* and *alg-2* causes embryonic lethality with a predominant arrest in the morphogenetic process of elongation (Vasquez-Rifo et al., 2012). Disruption of *ago1* in *Drosophila* causes embryonic lethality with notable nervous system abnormalities (Kataoka et al., 2001). Loss of Dicer in Zebrafish results in abnormal gastrulation causing severe defects in brain development and organogenesis. Notably, injection of a miR-430 duplex into embryos considerably rescued neuronal defects, indicating an outstanding role of a single miRNA in early fish development (Giraldez et al., 2005). Mouse embryos lacking *Dicer1* or *Ago2* fail to undergo gastrulation, accompanied by a malformation of germ layers (Bernstein et al., 2003; Alisch et al., 2007). Moreover, this phenotype is recapitulated in animals bearing a deletion of *Dgcr8* (Wang et al., 2007). In plants, disruption of DICER-LIKE1 leads to embryonic arrest early in development, likely caused by a failure to prevent precocious differentiation events (Nodine and Bartel, 2010). Interpretation of these experiments should consider possible miRNA-independent functions of disrupted miRNA-pathway factors, as such roles have been demonstrated for Drosha, Pasha and Dicer (Chong et al., 2010; Macias et al., 2012; Gromak et al., 2013; Luhur et al., 2014; Rybak-Wolf et al., 2014; Cirera-Salinas et al., 2017; Kim et al., 2017; Marinaro et al., 2017). However, given that removal of different components in the miRNA-pathway causes highly similar phenotypes, it is strongly suggested that miRNA-deficiency underlies the observed defects.

Whereas miRNAs are collectively essential in animals, uncovering the functions of many individual miRNAs has been challenging (Miska et al., 2007; Park et al., 2012; Chen et al., 2014). This is in part due to redundancy (Ge et al., 2012), most notably among miRNAs that fall into so-called families and share targeting specificity (Alvarez-Saavedra and Horvitz, 2010), but also redundancy with other repressive mechanisms that contribute to the output of gene regulatory networks (acting either at the RNA or protein levels). For example, in *Drosophila*, the near-complete loss of miRNA-mediated repression is tolerated to a large extent if general metabolism is simultaneously slowed down (Cassidy et al., 2019). This suggests that under conditions of slow developmental tempo, the timely downregulation of targets or the establishment of the right protein levels, to which miRNAs contribute, might be achieved through compensatory mechanisms. In a number of cases, the contribution of miRNAs has been revealed only in sensitized genetic backgrounds or upon environmental perturbation, such as exposure to extreme temperatures or pathogen stress. This suggests that the activity of some miRNAs is redundant with other factors that are influenced by environmental conditions (Hornstein and Shomron, 2006; Brenner et al., 2010; Ebert and Sharp, 2012; Cassidy et al., 2013, 2016, 2019; Siciliano et al., 2013; Ren and Ambros, 2015; Ilbay and Ambros, 2019). It is also likely that the function of many miRNAs has not been uncovered, even if they play non-redundant roles, because they are often expressed with very high spatial and temporal specificity and may function predominantly in specialized cell types within complex multicellular organisms (Alberti and Cochella, 2017).

## miRNA Production and Its Regulation

Multicellular organisms have taken advantage of the potential of miRNA-mediated regulation employing multiple mechanisms to control the production of miRNAs in distinct cell-types, under varying conditions, or during different developmental stages. miRNA biogenesis begins with transcription of a primary transcript by RNA polymerase II (Figure 2). Transcriptional regulation will thus determine whether a miRNA will be produced in the first place, and in general miRNA abundance correlates well with the rates of primary-miRNA transcription (Reichholf et al., 2019). At the organismal level, there is evidence that for most miRNAs, transcriptional control is the main determinant of cell-type specificity (Alberti et al., 2018). However, the maturation of primary miRNAs, initially by the Microprocessor in the nucleus and later by Dicer in the cytoplasm (Figure 2), can also be regulated, contributing significantly to the dynamics of accumulation and steady-state abundance of different miRNAs (Conrad et al., 2014). Moreover, it became evident that regulation of miRNA decay plays an important role in determining the functional levels of individual miRNAs (Zhou et al., 2018; Reichholf et al., 2019).

Transcription of each pri-miRNA is under the control of specific transcription factors and enhancers that outline their expression patterns. For example, an elaborate transcriptional mechanism determines the expression of the miRNA *lsy-6* in a single neuron in *C. elegans* (Cochella and Hobert, 2012). In contrast, post-transcriptional regulation of miRNA biogenesis is achieved at two different levels: (i) globally, by controlling the shared core machinery that produces most miRNAs, e.g., modulating levels or activity of the Microprocessor, and (ii) at the level of individual miRNAs, exploiting unique sequence and structural features of diverse miRNA precursors which impact interaction with the biogenesis machinery. Such interactions are often facilitated by various RNA binding proteins (RBPs).

Recent advances in the use of metabolic labeling of RNA have enabled kinetic studies of different steps during the life of a miRNA, and provided insight into how the rates of these steps vary for distinct miRNAs (Reichholf et al., 2019). An in-depth analysis in *Drosophila* S2 cells revealed that the rates of mature miRNA biogenesis range from 17 to >200 molecules/minute/cell. However, the loading into Argonaute is significantly slower and in fact represents a kinetic bottleneck in the production of functional miRISC. As a consequence, a large fraction of the miRNA duplexes produced by Dicer (approximately 40%) is degraded before loading. Other reports found only <10% of cellular miRNAs are bound by Argonaute (Janas et al., 2012; Stalder et al., 2013). This seemingly wasteful strategy ensures specific loading of Argonaute with miRNAs, which compete against other abundant duplex RNAs originating from tRNAs, rRNAs, snRNAs, or snoRNAs (Reichholf et al., 2019). Consistently, Argonaute binding is a better indicator of the inhibitory potential of a miRNA than its overall concentration (Flores et al., 2014). For this specificity mechanism to be effective, Argonaute levels need to be limited. This is achieved in large part through the relatively short half-life of empty Argonaute, which is efficiently ubiquitinated and degraded (Diederichs and Haber, 2007; Derrien et al., 2012; Janas et al., 2012; Martinez and Gregory, 2013; Smibert et al., 2013; Kobayashi et al., 2019; Reichholf et al., 2019; Bose et al., 2020).

Another important contributor to miRNA abundance is their stability. While overall miRNAs are among the most stable cellular RNAs, individual species can range in stability from minutes to more than a day (Reichholf et al., 2019). Rates of decay can be modulated by external stimuli to remodel the miRNome, as is the case for light-regulated retinal miRNAs (Krol et al., 2010). In general, neuronal miRNAs in mammals appear to have a high turnover rate compared to other cell types, and their abundance can be rapidly regulated in connection with neuronal activity (Krol et al., 2010). Together with the biogenesis rates, the different decay rates determine the steady-state abundance of miRNAs, such that two miRNAs can reach the same abundance through contrasting strategies: a miRNA with slow biogenesis and slow decay can accumulate to the same concentration as one with fast biogenesis and fast decay. The first case will result in a stable concentration that is less likely to change upon small perturbations, while the second is energetically more costly but provides potential for dynamic regulation. Such different strategies for accumulation have important consequences for miRNA function, and further investigation of how these different rates come about will provide critical insight. Nevertheless, this quantitative understanding already provides a framework for determining the ability of miRNAs to reach the necessary cellular concentrations to execute their repressive functions.

Effective repression by miRISC requires a high concentration of a miRNA relative to its target (Ameres and Zamore, 2013). To achieve this, synthesis of a number of miRNAs begins long before the onset of their repressive function. For example, the miRNA *lsy-6* in *C. elegans*, which functions in a sensory neuron by repressing the transcription factor COG-1 (Johnston and Hobert, 2003), is produced in the mother of the sensory neuron (Cochella and Hobert, 2012). Transcription of *cog-1* begins in the postmitotic neuron several hours later in a cell that has high concentration of *lsy-6* (Cochella and Hobert, 2012). This likely contributes to the ability of *lsy-6* to act as a genetic switch by completely preventing COG-1 expression (Johnston and Hobert, 2003; Cochella and Hobert, 2012). Another example of this is the essential *C. elegans* miRNA *let-7*, which is required for transition of the last larval stage to adulthood, yet starts being transcribed in the first larval stage (Martinez et al., 2008; Van Wynsberghe et al., 2011). Taking into account the relative dynamics of accumulation of a given miRNA and its target/s offers helpful insight for understanding the contribution of that miRNA to specific cellular processes.

These are the general forces that determine whether a miRNA is made and if so, to what level it accumulates relative to its targets. The core mechanisms that control each of these steps have been studied in great detail in different cell-based systems. Multicellular organisms take advantage of various ways to adjust these mechanisms to generate the necessary spatio-temporal specificity and the dynamics of miRNA expression that support development and homeostasis.

### Drosha/DGCR8 and Dicer: The Gatekeepers of miRNA Production

Processing of primary-miRNA transcripts (pri-miRNAs) initiates co-transcriptionally (Lee et al., 2003; Lee Y. et al., 2004; Morlando et al., 2008; Ballarino et al., 2009). In *Drosophila*, Pasha/DGCR8 can associate directly with RNA pol II via its phosphorylated C-terminal domain, linking transcription with the first step of miRNA maturation (Church et al., 2017). Interaction of DGCR8 and Drosha with pri-miRNAs occurs via recognition of a hairpin secondary structure flanked by single-stranded RNA and leads to cleavage by Drosha to release a precursor hairpin (Lee et al., 2003; Han et al., 2004; Sohn et al., 2007). The efficiency and the accuracy of this reaction are crucial determinants of miRNA abundance and targeting specificity, respectively. Because miRNAs bind their targets primarily through nucleotides 2–8 relative to the 5′ end of the miRNA (the “seed” sequence), a change in cleavage site that affects the 5′ end of a miRNA even by a single nucleotide can drastically alter target specificity of that miRNA. The choice of cleavage site by Drosha is crucial for setting the 5′ end of both miRNA arms: the 5p arm directly and the 3p indirectly by determining the register for the subsequent cleavage by Dicer (Auyeung et al., 2013; Partin et al., 2017; Kwon et al., 2019). Interestingly, recent mapping of Drosha cleavage sites with single nucleotide resolution in human cell lines (HEK293T and HeLa cells), revealed that some miRNAs undergo alternative processing resulting in different 5′ and 3′ ends (Kim et al., 2017). This has the potential to further diversify the miRNA-repertoire.

Once a pri-miRNA is processed to a precursor hairpin (pre-miRNA) in the nucleus, it will be exported and further cleaved in the cytoplasm by Dicer (Bernstein et al., 2001; Grishok et al., 2001; Ketting et al., 2001; Lee et al., 2002). Consistent with its evolutionary origin before the appearance of miRNAs (Mukherjee et al., 2013), Dicer has a wider range of substrates than the Microprocessor – it can cleave RNA duplexes of any length, as long as they have a 2-nt 3′ overhang (and in some cases even blunt-ended duplex RNA). The domain structure of Dicer serves as a molecular “ruler,” such that the two RNAse III active sites are positioned at a defined length from the 3′ overhang of the pre-miRNA and determine the length of the mature small RNA (MacRae et al., 2006, 2007). Dicer cooperates with other RNA binding proteins, PACT or TRBP in humans, and Loquacious in *Drosophila* (Treiber et al., 2019). This interaction can affect not only the efficiency of the processing, but also the length of the mature miRNA produced (Lee et al., 2006; Chakravarthy et al., 2010; Fukunaga et al., 2012; Lee and Doudna, 2012; Zhu et al., 2018). Therefore, together with Drosha, Dicer defines not only the abundance but also the ends of the mature miRNAs that will be loaded into Argonaute.

Because of its broader activity, Dicer processes not only pre-miRNAs, but also different endogenous and foreign duplex RNAs to produce other classes of short interfering RNAs (siRNAs). Whereas *Drosophila* has two different Dicer proteins, one specialized for miRNA (*Dcr-1*) and the other for siRNA biogenesis (*Dcr-2*) (Lee Y. S. et al., 2004), many other animals have a single type of Dicer. The involvement of this enzyme in two different pathways creates a bottleneck that in some animals leads to competition between the two different types of small RNA precursors. In *C. elegans* for example, downregulation of the endo-siRNA pathway results in an increase of miRNA-biogenesis, whereas induction of exogenous RNAi competes with both endo siRNA and miRNA production. This suggests that, at least in some contexts, Dicer can be limiting for small RNA production (Zhuang and Hunter, 2011).

Processing by the Microprocessor and by Dicer is subjected to diverse regulatory mechanisms. These can either affect Drosha and DGCR8 or Dicer themselves to broadly impact the biogenesis of multiple miRNAs, or specifically regulate the maturation of individual miRNAs, typically through the action of RNA binding proteins (RBPs) that recognize unique features of primary and precursor miRNAs.

### Global and miRNA-Specific Regulation Determine the Rates of miRNA Production

Pri-miRNAs share some broad structural features, but they can differ substantially in the length of their stems, the sequences in their loops, and the sequences flanking the hairpin, posing a challenge for efficient and specific processing by the Microprocessor. Indeed, not all wild-type pri-miRNAs are optimal targets for Drosha or DGCR8 binding, resulting in a broad range of processing efficiencies (Han et al., 2006; Auyeung et al., 2013; Fang and Bartel, 2015; Kim et al., 2017), and different degrees of sensitivity to the presence of co-factors like for example DGCR8’s co-factor, the iron-containing porphyrin heme (Partin et al., 2017; Nguyen et al., 2018). A number of sequence motifs that increase the specificity and efficiency of processing by the Microprocessor have been identified; for instance, apical elements in the hairpin, most prominently a UGU motif (Auyeung et al., 2013) and the mGHG-motif on the basal side, which seems to be a key determinant of cleavage site selection in many pri-miRNAs (Kwon et al., 2019). Highlighting the importance of the pri-miRNA sequence and structure for its correct processing, a single nucleotide change in the apical loop of pri-mir-30c-1 found in some patients with breast and gastric cancer results in increased processing and thus higher miR-30c-1 abundance. This was attributed to enhanced binding of SRSF3, a protein of the SR family that promotes Microprocessor cleavage (Fernandez et al., 2017).

The diversity in pri- and pre-miRNA sequences means that different steps might be rate-limiting for individual precursors, and this has enabled the evolution of additional regulatory mechanisms. A number of individual miRNAs are subjected to unique modes of regulation via specific interactions with different RNA-binding proteins (RBPs) (Choudhury et al., 2013; Treiber et al., 2017; Kooshapur et al., 2018; Michlewski and Cáceres, 2018; Downie Ruiz Velasco et al., 2019). A recent mass spectrometry-based screen using various human cell line lysates identified numerous RBPs that impact processing of subsets of miRNA-precursors and ultimately the expression of target mRNAs (Treiber et al., 2017). The binding specificity of many of these seems to be determined by the terminal loop, in which the RNA is single-stranded and typically exposed for contact with other proteins (Choudhury and Michlewski, 2012; Treiber et al., 2017). However, interestingly a few RBPs also bind the stem of specific miRNA hairpins (Treiber et al., 2017). A complementary *in silico* approach using published eCLIP-data has also identified a number of novel RBP:pre-miRNA interactions affecting processing of specific miRNAs (Nussbacher and Yeo, 2018).

Some RBPs have been shown to recruit terminal nucleotidyl transferases, most commonly uridyl transferases or TUTases (Hagan et al., 2009). Post-transcriptional modifications of precursor or mature miRNAs can impact the kinetics of biogenesis or silencing. Uridylation of mature miRNAs by TUTases tends to promote decay. However, uridylation of pre-miRNAs may either promote decay or have stabilizing effects and affect further processing by Dicer, depending on the 3′ end structure of the precursor (Thornton et al., 2014; Bortolamiol-Becet et al., 2015; Kim et al., 2015; Reimão-Pinto et al., 2015). Terminal miRNA modification by TUTases occur in Bilateria as well as in more basal animal clades such as Cnidaria and Porifera. In fact, TUTases acting on small RNAs were already present in the last common ancestor of all animals, underscoring the fundamental involvement of these mechanisms in miRNA biology (Modepalli and Moran, 2017).

The general activities of Dicer and the Microprocessor can also be regulated, with global consequences for miRNA output. A common regulatory mechanism is through post-translational modifications of the enzymes themselves, or their cofactors (Treiber et al., 2019). Regulation of Dicer activity for example is affected by phosphorylation of its binding partner TRBP, resulting in increased stability of the Dicer-TRBP complex and enhanced pre-miRNA processing activity (Paroo et al., 2009; Xu et al., 2015; Warner et al., 2016). Dicer itself can be phosphorylated on two conserved residues, resulting in its nuclear translocation and inhibition in worms, mice and humans (Drake et al., 2014). This inhibitory mechanism has been implicated in the reduction of mature miRNA levels in the *C. elegans* germline, contributing to precise gene expression changes in the oocyte-to-embryo transition (Drake et al., 2014). In general, post-translational modifications may serve to integrate diverse cellular signaling pathways with the production of miRNAs.

Moreover, levels of the processing machinery can vary across different cell types or under different conditions, also impacting the global miRNA output. A survey of Drosha expression levels across different mouse tissues revealed differences in the range of 4–10-fold, with the brain having the highest and the liver the lowest level of Drosha mRNA and protein (Sperber et al., 2014). Lower Drosha expression enhances the inherent differences in processing efficiency of different pri-miRNAs, resulting in the deregulation of subsets of miRNAs with specific properties (Sperber et al., 2014). The contribution of some of these mechanisms to the development and physiology of animals remains to be tested. Nevertheless, the dysregulation of the miRNA biogenesis machinery has been associated with diverse forms of cancer, typically resulting in a global repression of miRNA maturation (Lin and Gregory, 2015).

The final step for producing a functional miRISC is the loading of a miRNA into Argonaute. This seems to be the rate-limiting step in a number of contexts (Diederichs and Haber, 2007; Janas et al., 2012; Reichholf et al., 2019; Bose et al., 2020), likely related to the fact that loading does not simply reflect binding of an RNA to Argonaute, but follows a number of steps that require assistance from other factors, including chaperones and energy from ATP (Kobayashi and Tomari, 2016). As with every other step of miRNA biogenesis, miRNA loading can also be affected by specific features of the miRNA duplex, such as presence of a 5′ phosphate, identity of the 5′ terminal nucleotide and stability of the duplex. These features also determine which of the two duplex strands is preferentially loaded on Argonaute and acts as a mature miRNA; the opposite strand, or miRNA^∗^, is typically rapidly degraded. In addition, specific RNA binding proteins that impact the loading on Argonaute either positively or negatively have been described. For example, TDP43 disrupts loading of miR-1 and miR-206, two muscle-specific miRNAs (King et al., 2014), while hnRNPD0 supports loading of let-7b (Yoon et al., 2015).

The effects of all these regulatory mechanisms at the organismal level can in principle have two different types of consequences. On the one hand, post-transcriptional regulation of miRNA biogenesis may be used to encode temporal or spatial information in a developing animal. Such is the case of LIN28, which acts as a conserved post-transcriptional repressor of let-7-family miRNA biogenesis (Moss et al., 1997; Yang and Moss, 2003; Heo et al., 2008; Newman et al., 2008; Viswanathan et al., 2008). LIN28 recruits TUTases that ultimately prevent Dicer-recognition and promote decay via the exonuclease Dis3L2 (Hagan et al., 2009; Chang et al., 2013). Release of LIN28-mediated repression of let-7 maturation provides a temporal switch in different contexts during progression of animal development (Thornton and Gregory, 2012).

However, most of the RBPs that affect biogenesis and loading are ubiquitously or very broadly expressed, suggesting that they may rather contribute to achieving homeostatic levels of specific miRNAs. This could be necessary to compensate for individual miRNA features that make them better or worse substrates of the biogenesis machinery. It is also possible that some of these RBPs have different expression levels or modifications in different tissues that might directly contribute to the spatio-temporal specificity of miRNA production. Another possibility is that some regulators are used to restrict the production of miRNAs to specific sub-cellular localizations. An extreme example of this is the synapse-specific maturation of miRNAs by Dicer upon neuronal activation (Sambandan et al., 2017).

## Silencing Mechanism – Variations on a Theme

**FIGURE 3:**
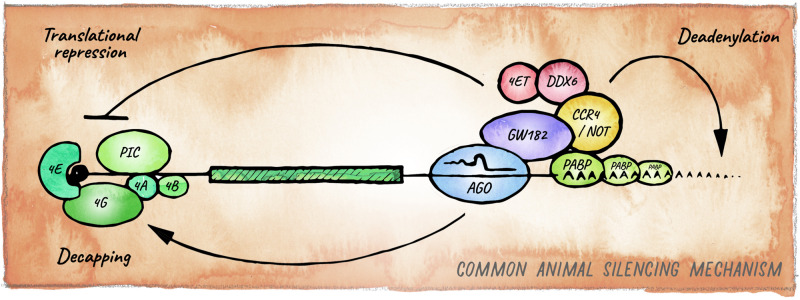
Canonical miRNA-silencing mechanism in animals. miRNAs elicit repression of target genes usually through a combination of translational repression and promotion of mRNA decay. Argonaute is guided by a miRNA to a cognate target mRNA and tethers GW182, forming the core of the most common animal miRISC. GW182 interacts with PABP and recruits the deadenylase complexes CCR4-NOT or Pan2-Pan3 (not shown), leading to deadenylation, decapping and ultimately exonucleolytic decay. Inhibition of translation occurs mainly at the initiation step by interfering with assembly or activity of eIF4F, via eIF4E-T, and DDX6.

The ancestral mechanism of small RNA guided effector proteins involves irreversible destruction of targeted nucleic acids by cleavage (Park and Shin, 2014; Moran et al., 2017). This miRNA mode of action, which is usually accompanied by near-perfect target complementarity, is prevalent in plants (Voinnet, 2009) and also observed in basal metazoan lineages like Cnidaria (Moran et al., 2014; Mauri et al., 2017). Bilaterian animals on the other hand, predominantly employ a mechanism that relies on partial base pairing between the “seed” region of a miRNA (nucleotides 2–8) and sequences typically in the 3′ UTR of mRNAs. Recruitment of miRISC in bilaterians usually results in the downregulation of protein output through a combination of translation inhibition and target mRNA decay (Jonas and Izaurralde, 2015; Bartel, 2018; Figure 3).

Appearance of the slicing-independent, seed-based mechanism laid the foundation for functional diversification of miRNA biology in animals. Compared to a mode of target recognition that involves full sequence complementarity, a mechanism that requires only partial base-pairing has the potential to greatly increase the target repertoire of any miRNA. This likely expanded the overall potential of miRNA-mediated repression in bilaterian animals, enhancing the connectivity of gene regulatory networks, and contributing to cell-type diversification and acquisition of morphological complexity during evolution (Peterson et al., 2009; Moran et al., 2017).

### The Metazoan Seed-Based Mechanism

The best understood mechanism of miRNA-mediated repression in animals relies on a protein of the GW182 family, which together with Argonaute forms the core of what is considered the canonical miRISC. Members of the GW182 protein family (e.g., GW182 in *Drosophila*, AIN-1/2 in *C. elegans* and TNRC6 in humans) are rapidly evolving but share Gly-Trp repeats that bind to conserved pockets in Argonaute proteins (El-Shami et al., 2007; Zhang et al., 2007; Eulalio et al., 2008; Pfaff et al., 2013). Through other domains, GW182 proteins recruit RNA-processing factors, repressing translation as well as enhancing mRNA turnover (Chekulaeva et al., 2011; Fabian et al., 2011; Kuzuoğlu-Öztürk et al., 2012; Huntzinger et al., 2013). The precise mechanism for translational repression remains to be resolved; however, the emerging consensus is that it involves inhibition of cap-dependent translation initiation via interaction with eIF4F (Pillai et al., 2004; Mathonnet et al., 2007; Zdanowicz et al., 2009; Ricci et al., 2013; Fukaya et al., 2014). GW182 proteins also recruit the PAN2-PAN3 and CCR4-NOT deadenylase complexes, ultimately triggering mRNA decay (Behm-Ansmant et al., 2006; Guo et al., 2010; Braun et al., 2013; Eichhorn et al., 2014; Jonas and Izaurralde, 2015; Kuzuoğlu Öztürk et al., 2016; Niaz and Hussain, 2018; Figure 3). GW182 has been proposed to promote the formation of phase-separated condensates containing miRISC and target mRNAs, increasing the local concentration of deadenylases and other factors for efficient repression (Sheu-Gruttadauria and MacRae, 2018). GW182 is also present in Nematostella, where it is able to interact with Argonaute and the CCR4-NOT complex, suggesting that this mechanism originated in the last common ancestor between Cnidaria and Bilateria (Mauri et al., 2017). Experimental uncoupling of translational inhibition and mRNA decay has proven challenging, as the two processes are intimately linked (Subtelny et al., 2014; Radhakrishnan and Green, 2016). Still, analyses of the dynamics of miRNA-mediated silencing in zebrafish early embryos and in mammalian cells in culture, revealed that miRNAs first repress translation initiation and then induce mRNA decay (Bazzini et al., 2012; Djuranovic et al., 2012). The relative contribution of these two mechanisms to biological function remains a matter of debate (Eichhorn et al., 2014), but as we discuss below multiple pieces of evidence indicate that this may be determined by the cellular context and miRISC composition.

**FIGURE 4:**
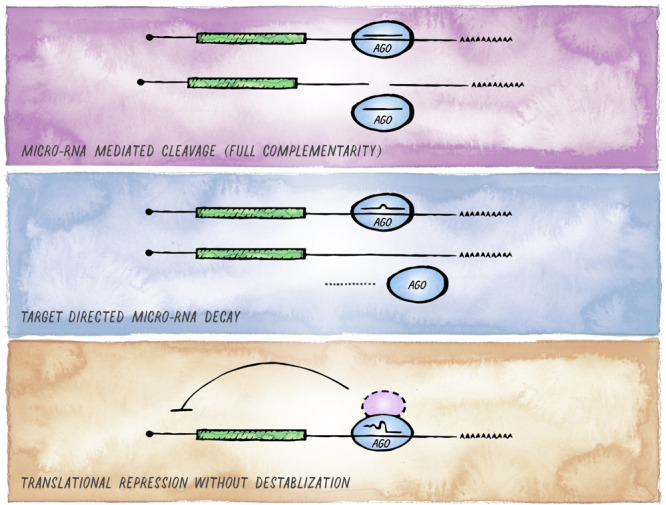
Alternative outcomes of miRNA-mediated targeting. In addition to the common miRISC effects on translation and stability of target mRNAs, other functional outcomes of miRISC binding to an mRNA are possible. **(A)** Full sequence complementarity results in Ago-mediated target cleavage, a mechanism that resembles the mode of action commonly employed in cnidaria and plants. **(B)** Target-mediated miRNA degradation is induced by interaction with targets through extensive pairing, in particular extending to the miRNA 3′ end. **(C)** Recruitment of Argonaute in the absence of GW182 results in inhibition of translation without affecting mRNA stability (likely involving alternative co-factors).

Despite the conserved nature of the canonical miRNA targeting mechanism, there is an increasing number of examples highlighting context-dependent variability in the mode and functional consequence of miRISC targeting (Figure 4). This variability can arise from two different mechanistic sources. First, miRISC can have non-canonical composition in different cell types or distinct developmental stages (e.g., differential presence or abundance of GW182 and other proteins that act as facultative Argonaute interactors). In metazoans, such variations are observed most notably between soma and germline, and we expand on this in the next section. Second, as perfect target complementarity is not a prerequisite, there are different possible miRNA:mRNA interaction modes (Lal et al., 2009; Shin et al., 2010; Chi et al., 2012). Whereas the majority of miRNA binding sites are located in the 3′ UTR and involve pairing to the seed region of a miRNA, these features can differ with varying functional consequences; from efficiency of silencing depending on the relative location of miRNA-binding sites within the target mRNAs (Grimson et al., 2007; Forman and Coller, 2010; Zhang K. et al., 2018); to degradation of the miRNA itself, if target pairing extends to the 3′ end of the miRNA (Ameres et al., 2010; Cazalla et al., 2010; De la Mata et al., 2015; Bitetti et al., 2018; Ghini et al., 2018; Sheu-Gruttadauria et al., 2019).

### Variations in miRISC Composition and Functional Outcome

The mode of miRNA targeting and the effects that different RISC complexes will exert on their targets vary significantly across different eukaryotic clades. Even within a single animal, the composition and functional consequences of miRISC can differ in specific biological settings. The first evident variation is in the Argonaute component itself, e.g., *C. elegans* evolved more than 25 Argonaute proteins (Youngman and Claycomb, 2014). ALG-1 and ALG-2 are the main miRNA-related Argonautes. They function redundantly to a large degree, and only loss of both simultaneously causes penetrant embryonic lethality (Vasquez-Rifo et al., 2012). However, they do display some functional differences which may stem from differences in expression patterns and levels (ALG-2 appears to be dominantly expressed throughout embryogenesis) as well as in miRNA-binding preference (Vasquez-Rifo et al., 2012; Brown et al., 2017). Interestingly, another Argonaute, ALG-5, has recently been implicated in miRNA-mediated silencing in the worm. ALG-5 is expressed in the germline, where it associates effectively with only a subset of miRNAs and plays a role in gametogenesis (Brown et al., 2017). Insects such as cockroaches, from a basal clade, also have two partially redundant Argonautes for miRNA mediated silencing (Rubio et al., 2018). However, subsequent specialization took place in more derived clades like *Drosophila*, which encodes one Argonaute for siRNA and another one for miRNA-mediated silencing (Förstemann et al., 2007; Iwasaki et al., 2009).

Most of the variation in outcome of miRISC binding to an mRNA stems from differential association of Argonaute with other proteins. This seems to primarily affect the relative contribution of translation inhibition vs. mRNA destabilization. In particular, the larger differences appear to occur between the germline or early embryo versus somatic tissues. In *C. elegans*, the prevalent form of miRISC in the germline has been shown to lack the GW182 proteins AIN-1 and AIN-2 and instead associates with P-granule constituents. The resulting recruitment of target mRNAs to P-granules inhibits protein production but does not promote mRNA decay (Dallaire et al., 2018). This has been proposed to protect maternal mRNAs whose translation products are not beneficial in the germline but are required in the early embryo for robust development. Consistent with these observations, *C. elegans* embryos tolerate mutations that severely impede association of Argonaute with AIN-1/2. Despite Argonautes being essential for embryonic development of *C. elegans* (Vasquez-Rifo et al., 2012), AIN-1/2 play a secondary role at this stage (Jannot et al., 2016). However, the interaction between Argonaute and the GW182 orthologs is necessary for post-embryonic development (Jannot et al., 2016).

GW182-independent function of miRISC has also been observed in other contexts. For instance, in *Drosophila* S2 cells, induction of mitogenic signaling via serum withdrawal results in formation of an ER-associated, GW182-independent miRISC. This complex is a potent inhibitor of translation but has no effect on mRNA deadenylation and decay (Wu et al., 2013). In line with this, depletion of GW182 in *Drosophila* S2 cells abolished miRNA-dependent deadenylation but had practically no effect on translation repression (Fukaya and Tomari, 2012). The diversity in miRNA mediated silencing mechanisms provides organisms with enhanced capacity for gene regulation, while posing additional challenges for a complete understanding of miRNA functions *in vivo*.

Animals like *C. elegans* have adopted variant miRNA activity in the germline, yet other animals seem to have evolved ways to dampen or abolish germline miRNA activity altogether. In *Drosophila* oocytes, Ago-1 is present at very low levels and only increases upon maternal to zygotic transition (Luo et al., 2016), coinciding with the production of a miRNA cluster involved in maternal mRNA decay (Bushati et al., 2008). Zebrafish zygotes also have low miRNA levels, with considerable accumulation starting around the blastula stage (Chen et al., 2005). At that time, miRNAs are also involved in the maternal to zygotic transition. Most notably, miR-430 is expressed at the onset of zygotic genome activation and promotes maternal mRNA clearance (Giraldez et al., 2006). The mouse germline also appears to lack essential miRNA functions, as depletion of DGCR8 results in normal oocytes that give rise to healthy offspring upon fertilization with wild-type sperm (Suh et al., 2010). In the absence of both maternal and zygotic DGCR8, zygotes still undergo normal pre-implantation development but then arrest prior to E6.5 (Suh et al., 2010). Clearly, miRNA-mediated regulation plays important roles in early animal development, yet the contribution and mechanisms of action in the developmental window around fertilization remain an active area of investigation (McJunkin, 2017).

miRNAs provide an outstanding way to confer specificity to a variety of repressor complexes. Different effector mechanisms may operate within one organism, or even within one cell, and may be dynamically regulated under different conditions. It will be interesting to find out how different modes of regulation are used in different cellular contexts within animal development and physiology.

## Innovation Through Expansion of the miRNA Repertoire

**FIGURE 5:**
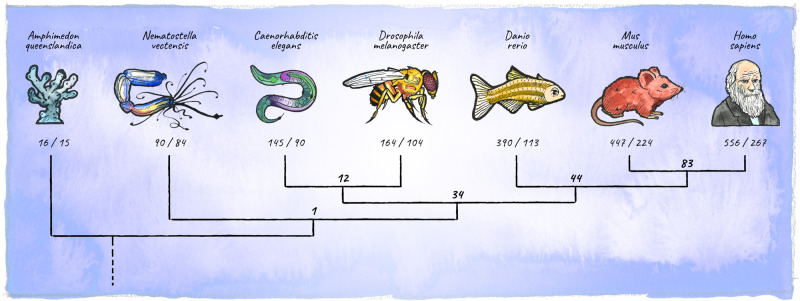
Diversification of the metazoan miRNA repertoire. Shown are numbers of individual miRNAs/miRNA-families (in bold) annotated with high confidence for various clades (https://mirgenedb.org, Fromm et al., 2020). Number of miRNA families present in the last common ancestors of branching clades are noted above the split. Exemplary organisms depicted are *Amphimedon queenslandica*, *Nematostella vectensis*, *Caenorhabditis elegans*, *Drosophila melanogaster*, *Danio rerio*, *Mus musculus*, and *Homo sapiens*. Data for bilateria is derived from Fromm et al. (2020), for *Amphimedon* and *Nematostella* numbers were retrieved from the work of Grimson et al. (2008); Moran et al. (2014), and Calcino et al. (2018).

In the course of animal evolution, the miRNA repertoire expanded drastically in conjunction with complexity (Hertel et al., 2006; Heimberg et al., 2008; Wheeler et al., 2009; Berezikov, 2011; Guerra-Assunção and Enright, 2012; Hertel and Stadler, 2015; Figure 5). Often, miRNAs arose in bursts coinciding with major organismal innovations, for example the emergence of vertebrates, or cortical expansion in primates (Peterson et al., 2009; Hertel and Stadler, 2015; Kosik and Nowakowski, 2018; Fromm et al., 2020). While the animal miRNA machinery originated in unicellular holozoans (Bråte et al., 2018), the most conserved animal miRNA, miR-100, first appeared in the last common ancestor of cnidarians and bilateria (Grimson et al., 2008). Organisms diverging early in eukaryotic evolution tend to have few, mostly non-conserved miRNA genes, indicating a high evolutionary flux in basal clades (Grimson et al., 2008; Cock et al., 2017). The availability of high-quality miRNA annotations in diverse genomes has recently upgraded our ability to place miRNAs into an evolutionary context (Berruezo et al., 2017; Cock et al., 2017; Wang et al., 2017; Ylla et al., 2017; Fromm et al., 2019, 2020). This opens up exciting new avenues, enabling connections between miRNA age, expression and function during development, as well as the emergence of specific features at the organismal level.

### Diversification of the miRNA Repertoire

A widespread view in the field is that miRNAs tend to be rapidly gained and lost in the course of evolution (Nozawa et al., 2010; Fromm et al., 2013; Meunier et al., 2013), although part of this has been recently challenged (Tarver et al., 2018). Nevertheless, a substantial number of miRNAs have acquired important functions and remain conserved. Because miRNA evolution is intimately connected to evolution of target genes (Zhao et al., 2015; Nozawa et al., 2016), conservation of miRNAs is observed primarily over the seed region, which determines targeting specificity. However, features beyond the seed can critically impact miRNA function, underscored by the observation that some mature miRNAs are conserved from the first to the last nucleotide across large evolutionary distances. For example, *let-7* has not accumulated a single mutation between humans and worms (Pasquinelli et al., 2000), or *mir-9a*, has remained identical between Drosophila, mouse, and human (Li et al., 2006).

miRNAs are much younger than protein coding genes (on average 169 Myr vs. 1195 Myr respectively), and many of them arose more recently, after the split of diverse phylogenetic groups. For instance, an estimated 46% of human miRNAs are primate-specific and 14% are human-specific (Patel and Capra, 2017). In general, miRNA genes evolve *de novo* from hairpin-structures located within introns or intergenic regions (Lu et al., 2008; Nozawa et al., 2010), which in turn likely originate through one of three proposed models: (i) inverted gene duplication of a gene that will subsequently become target of the miRNA (Allen et al., 2004); (ii) transposon-insertion followed by derivatization (Li et al., 2011; Qin et al., 2015); and (iii) spontaneous evolution out of random sequences (De Felippes et al., 2008). However, the majority of functionally important miRNAs arose from duplications of existing miRNAs (Kim and Nam, 2006; Carthew and Sontheimer, 2009; Berezikov, 2011).

Diversification of the miRNA repertoire occurs both through the addition of new miRNAs and the addition or change in targets. An improved understanding of the latter will require better knowledge of what are the biologically meaningful targets of any given miRNA. Whereas for a few miRNAs we know of a number of experimentally well-defined targets, for most miRNAs we rely on computational prediction algorithms. Because such prediction tools yield numerous false positives, substantial experimental validation of targets will be necessary for a deeper understanding of how miRNA-target interactions change over time (Fridrich et al., 2019).

### miRNA Families

Duplication followed by sequence diversification of miRNAs can lead to target diversification, changes in expression pattern, and pronounced increases in dose (Luo et al., 2018). In many cases, if the seed sequence is retained, miRNA gene duplication marks the birth of a (homo-seed) miRNA family. Members of a family function largely redundantly on a shared set of target mRNAs. In many cases the full extent of the function of miRNA family becomes apparent only upon removing all members (Miska et al., 2007; Alvarez-Saavedra and Horvitz, 2010; Parchem et al., 2015). Curiously, most animal miRNAs whose loss of function causes severe defects, occur in families with sometimes extreme copy numbers. In *C. elegans*, two miRNA families are required for completion of embryonic development: the MIR-36 family (8 members in *C. elegans*, 29 members in the closely related *C. briggsae*) and MIR-100 family (miR-51-56 in the worm) (Alvarez-Saavedra and Horvitz, 2010). In zebrafish, MIR-430, which plays an outstanding role during embryonic development (Giraldez et al., 2005), has evolved at least 57 family members (according to mirgenedb2.0) located within a 16 kb region on chromosome 4. Mouse embryos lacking the miR-290-295 and miR-302-367 clusters (mammalian miR-430 homologs) arrest early in embryogenesis, with defects that are only partially recapitulated if one or the other family is removed (Medeiros et al., 2011; Parchem et al., 2015).

Underscoring the biological significance of miRNA duplication followed by organization into families, there seems to be an expansive trend among miRNAs with important biological functions. Potential reasons include (i) increasing the dose of mature miRNA to enhance efficient target silencing, or (ii) evolutionary robustness and flexibility, e.g., mutations in one family member are not immediately detrimental, and individual copies can be further diversified in sequence or in expression pattern. Different family members share identical seed sequences, and in some cases, paralogous miRNAs are also conserved around nucleotides 13–17, which underscores the contribution of these positions to efficient targeting (Wee et al., 2012; Schirle et al., 2014; Sheu-Gruttadauria and MacRae, 2017). However, in other cases family members differ in sequences beyond the seed, which can affect targeting properties (Broughton et al., 2016; Brancati and Großhans, 2018; Zhang S. et al., 2018) but also biogenesis or loading efficiency as discussed above. Thus, while miRNA family members appear largely redundant, individual members can acquire specific functions (Abbott et al., 2005; Alvarez-Saavedra and Horvitz, 2010; Brancati and Großhans, 2018; Zhang S. et al., 2018).

A recent analysis found that out of 352 human miRNAs that are conserved among vertebrates, 207 (58.8%) are duplicates and 125 (35.5%) are homo-seed family miRNAs (Luo et al., 2018). miRNAs in families differ from singletons in their evolutionary dynamics and functional roles, with family miRNAs tending to be: (i) older than singletons, (ii) more conserved at the sequence level than singletons, (iii) enriched for diverse expression in distinct tissues, (iv) broader in target range, and (v) implicated in more diseases. These functional correlations are still significant, albeit less pronounced, when family vs. singleton miRNAs are stratified by age, with older miRNAs tending to contribute more to disease, target more genes, and being expressed in significantly more tissues (Patel and Capra, 2017). In addition, the efficiency of target repression correlates with degree of conservation as well as evolutionary age for many miRNAs (Luo et al., 2018).

The expansion of metazoan miRNAs was likely one of the factors that contributed to the evolution of complexity in present-day animals. In this context, *de novo* evolution but also miRNA duplication followed by sequence diversification played an important role laying the foundation for miRNA families. Among others, family membership and evolutionary age of miRNAs coincides with functional trends, offering a useful context for elucidating the contribution of miRNAs to animal development and homeostasis. Nonetheless, clade-specific singleton miRNAs also provide a source of innovation. For example, miR-791 originated in a class of nematodes called Chromadorea, and at least in *C. elegans* it acquired an important function in its CO_2_-sensing neurons (Drexel et al., 2016). Similarly, *lsy-6* originated in the last *Caenorhabditis* common ancestor and plays an essential role in sensory neuron diversification in *C. elegans* (Johnston and Hobert, 2003).

## Concluding Remarks

Since the discovery of miRNAs, the field has made tremendous progress toward understanding the molecular mechanisms of biogenesis and action of this versatile class of repressors. The vast majority of these mechanistic studies have been performed in cell culture models, in which biochemical approaches are feasible. This has given us detailed snapshots of the possible roles of miRNAs at the molecular and cellular levels. At the organismal level however, most of our understanding comes from genetics, either from manipulations of the miRNA biogenesis machinery or individual miRNAs. While it is clear that miRNAs are necessary for the correct development and function of multiple cell types, in most cases we do not understand the functionally relevant relationships of miRNAs and their targets, the consequences on gene regulatory networks, and how the effect on specific cell types impacts the organism. Given the broad implication of miRNAs in physiology and disease, a deeper mechanistic understanding of the roles of miRNAs within complex organisms is highly desirable. We expect that new technologies that enable this depth of analysis in animals will make this possible.

## Author Contributions

PD and LC wrote the manuscript. PD designed, drew, and painted the figures in ink and watercolor.

## Conflict of Interest

The authors declare that the research was conducted in the absence of any commercial or financial relationships that could be construed as a potential conflict of interest.
